# [*meso*-Tetra­kis(4-heptyl­oxyphen­yl)porphyrinato]nickel(II)

**DOI:** 10.1107/S1600536810041942

**Published:** 2010-10-23

**Authors:** Liang Chen, Hong-Bin Zhao, Yu-Jia Xie, De-Liang Yang, Bang-Ying Wang

**Affiliations:** a Department of Organic Chemistry, the College of Chemistry, Xiangtan University, Hunan 411105, People’s Republic of China; b Environmental Engineering, Dongguan University of Technology, Guangdong 523808, People’s Republic of China

## Abstract

In the title compound, [Ni(C_72_H_84_N_4_O_4_)], the four-coordinate Ni^II^ ion in the middle of the planar 24-membered porphyrin ring is located on a crystallograpic inversion center, with Ni—N distances of 1.946 (2)–1.951 (2) Å. The 4-heptyl­oxyphenyl groups are twisted with respect to the porphyrin mean plane, the dihedral angles being 88.5 (3) and 79.1 (2)°.

## Related literature

For related structures, see: Scheidt (1977 ▶); Maclean *et al.* (1996 ▶); Jentzen *et al.* (1996 ▶). For background to porphyrins and metalloporphyrins, see: Kozaki *et al.* (2007 ▶); Kuciauskas *et al.* (1996 ▶); Suslick *et al.* (2005 ▶); Liu *et al.* (1985 ▶); Gross & Ini (1999 ▶); Wasielewski *et al.* (1993 ▶).
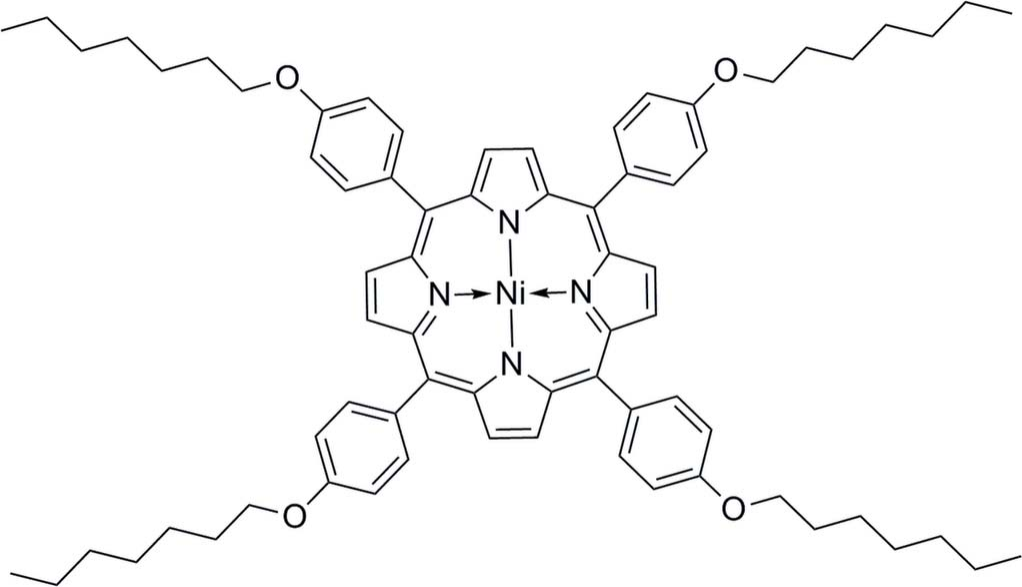


## Experimental

### 

#### Crystal data

[Ni(C_72_H_84_N_4_O_4_)]
*M* *_r_* = 1128.14
Monoclinic, 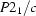

*a* = 15.8843 (12) Å
*b* = 19.0602 (15) Å
*c* = 10.2398 (8) Å
β = 91.221 (2)°
*V* = 3099.5 (4) Å^3^
*Z* = 2
Mo *K*α radiation
μ = 0.37 mm^−1^
*T* = 185 K
0.21 × 0.16 × 0.07 mm

#### Data collection

Bruker APEX CCD area-detector diffractometer
Absorption correction: multi-scan (*SADABS*; Sheldrick, 2004 ▶) *T*_min_ = 0.927, *T*_max_ = 0.975
14663 measured reflections
5469 independent reflections
3578 reflections with *I* > 2σ(*I*)
*R*_int_ = 0.062

#### Refinement

*R*[*F* ^2^ > 2σ(*F* ^2^)] = 0.051
*wR*(*F* ^2^) = 0.114
*S* = 0.96
5469 reflections
369 parameters
H-atom parameters constrained
Δρ_max_ = 0.36 e Å^−3^
Δρ_min_ = −0.35 e Å^−3^

### 

Data collection: *SMART* (Bruker, 2002 ▶); cell refinement: *SAINT* (Bruker, 2002 ▶); data reduction: *SAINT*; program(s) used to solve structure: *SHELXS97* (Sheldrick, 2008 ▶); program(s) used to refine structure: *SHELXL97* (Sheldrick, 2008 ▶); molecular graphics: *SHELXTL* (Sheldrick, 2008 ▶); software used to prepare material for publication: *SHELXTL*.

## Supplementary Material

Crystal structure: contains datablocks global, I. DOI: 10.1107/S1600536810041942/fk2026sup1.cif

Structure factors: contains datablocks I. DOI: 10.1107/S1600536810041942/fk2026Isup2.hkl

Additional supplementary materials:  crystallographic information; 3D view; checkCIF report

## Figures and Tables

**Fig. 1.**
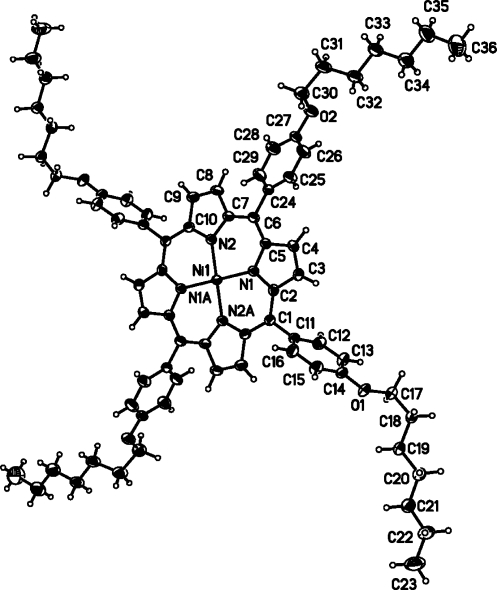
Molecular structure of (I), with the atom-labeling scheme and 50% probability displacement ellipsoids.

## supplementary crystallographic information

### Comment

Porphyrins and metalloporphyrins are researched in many aspects, such as electron and energy transfer (Kozaki *et al.*,2007; Kuciauskas *et al.*,1996), molecular recognition (Suslick *et al.*,2005), catalysts (Liu *et al.*,1985; Gross & Ini, 1999) or biomimetic models of photosynthetic systems (Wasielewski *et al.*,1993). In this paper, the structure of Nickel(II) *meso*-tetrakis[*p*-(heptyloxy)phenyl] porphyrinate is reported.

The 24-membered porphyrin moiety of the title compound is planar with a mean deviation of 0.045 (3) Å. The four-coordinate Ni^II^ ion is located at a crystallograpic inversion center, with Ni—N distances of 1.946 (2) to 1.951 (2) Å, in agreement with those found in other nickel porphyrin compounds (Scheidt,1977; Maclean *et al.*1996; Jentzen *et al.*1996.).

The *p*-pentyloxyphenyl groups are rotated at angles of 88.5 (3)° and 79.1 (2)° with respect to the porphyrin mean plane, due to steric hindrance with the pyrrole-H atoms of the macrocycle.

### Experimental

Single crystals were recrystallised from a dichloromethane solution at room temperature.

### Refinement

H atoms were placed in calculated positions (C—H = 0.95, 0.98 or 0.99 Å) and refined in riding mode, with *U*_iso_(H) = *xU*_eq_(C), where *x* = 1.5 for methyl and 1.2 for all other H atoms.

### Crystal data

**Table:**

[Ni(C_72_H_84_N_4_O_4_)]	*F*(000) = 1208
*M**_r_* = 1128.14	*D*_x_ = 1.209 Mg m^−^^3^
Monoclinic, *P*2_1_/*c*	Mo *K*α radiation, λ = 0.71073 Å
Hall symbol: -P 2ybc	Cell parameters from 1476 reflections
*a* = 15.8843 (12) Å	θ = 2.3–22.6°
*b* = 19.0602 (15) Å	µ = 0.37 mm^−^^1^
*c* = 10.2398 (8) Å	*T* = 185 K
β = 91.221 (2)°	Block, purple
*V* = 3099.5 (4) Å^3^	0.21 × 0.16 × 0.07 mm
*Z* = 2	

### Data collection

**Table:**

Bruker APEX CCD area-detector diffractometer	5469 independent reflections
Radiation source: fine-focus sealed tube	3578 reflections with *I* > 2σ(*I*)
graphite	*R*_int_ = 0.062
phi and ω scans	θ_max_ = 25.0°, θ_min_ = 1.7°
Absorption correction: multi-scan (*SADABS*; Sheldrick, 2004)	*h* = −18→16
*T*_min_ = 0.927, *T*_max_ = 0.975	*k* = −22→22
14663 measured reflections	*l* = −8→12

### Refinement

**Table:**

Refinement on *F*^2^	Primary atom site location: structure-invariant direct methods
Least-squares matrix: full	Secondary atom site location: difference Fourier map
*R*[*F*^2^ > 2σ(*F*^2^)] = 0.051	Hydrogen site location: inferred from neighbouring sites
*wR*(*F*^2^) = 0.114	H-atom parameters constrained
*S* = 0.96	*w* = 1/[σ^2^(*F*_o_^2^) + (0.0487*P*)^2^] where *P* = (*F*_o_^2^ + 2*F*_c_^2^)/3
5469 reflections	(Δ/σ)_max_ = 0.001
369 parameters	Δρ_max_ = 0.36 e Å^−^^3^
0 restraints	Δρ_min_ = −0.35 e Å^−^^3^

### Special details

**Table:**

Geometry. All e.s.d.'s (except the e.s.d. in the dihedral angle between two l.s. planes) are estimated using the full covariance matrix. The cell e.s.d.'s are taken into account individually in the estimation of e.s.d.'s in distances, angles and torsion angles; correlations between e.s.d.'s in cell parameters are only used when they are defined by crystal symmetry. An approximate (isotropic) treatment of cell e.s.d.'s is used for estimating e.s.d.'s involving l.s. planes.
Refinement. Refinement of *F*^2^ against ALL reflections. The weighted *R*-factor *wR* and goodness of fit *S* are based on *F*^2^, conventional *R*-factors *R* are based on *F*, with *F* set to zero for negative *F*^2^. The threshold expression of *F*^2^ > σ(*F*^2^) is used only for calculating *R*-factors(gt) *etc*. and is not relevant to the choice of reflections for refinement. *R*-factors based on *F*^2^ are statistically about twice as large as those based on *F*, and *R*- factors based on ALL data will be even larger.

### Fractional atomic coordinates and isotropic or equivalent isotropic displacement parameters (Å^2^)

**Table:**

	*x*	*y*	*z*	*U*_iso_*/*U*_eq_	
Ni1	1.0000	0.0000	0.0000	0.02268 (15)	
N1	0.93762 (14)	0.08453 (11)	0.0426 (2)	0.0234 (5)	
N2	0.90680 (13)	−0.05759 (11)	0.0596 (2)	0.0234 (5)	
O1	1.09497 (12)	0.46905 (9)	−0.07128 (19)	0.0323 (5)	
O2	0.51784 (12)	0.09270 (11)	0.4285 (2)	0.0408 (6)	
C1	1.03791 (18)	0.17651 (14)	−0.0219 (3)	0.0242 (7)	
C2	0.96102 (17)	0.15389 (14)	0.0243 (3)	0.0244 (7)	
C3	0.89751 (17)	0.20052 (15)	0.0693 (3)	0.0281 (7)	
H3	0.8985	0.2503	0.0646	0.034*	
C4	0.83648 (18)	0.16108 (15)	0.1191 (3)	0.0301 (7)	
H4	0.7866	0.1777	0.1583	0.036*	
C5	0.86001 (17)	0.08937 (14)	0.1025 (3)	0.0244 (7)	
C6	0.81084 (17)	0.03361 (15)	0.1406 (3)	0.0259 (7)	
C7	0.83248 (17)	−0.03534 (15)	0.1162 (3)	0.0267 (7)	
C8	0.78014 (18)	−0.09352 (15)	0.1457 (3)	0.0327 (8)	
H8	0.7260	−0.0914	0.1832	0.039*	
C9	0.82105 (17)	−0.15206 (16)	0.1109 (3)	0.0325 (8)	
H9	0.8013	−0.1989	0.1187	0.039*	
C10	0.90037 (17)	−0.13037 (14)	0.0595 (3)	0.0243 (7)	
C11	1.05300 (17)	0.25403 (14)	−0.0347 (3)	0.0252 (7)	
C12	1.01380 (19)	0.29119 (15)	−0.1341 (3)	0.0330 (8)	
H12	0.9778	0.2668	−0.1940	0.040*	
C13	1.02490 (18)	0.36291 (15)	−0.1501 (3)	0.0333 (8)	
H13	0.9960	0.3872	−0.2186	0.040*	
C14	1.07832 (17)	0.39857 (14)	−0.0654 (3)	0.0276 (7)	
C15	1.11814 (18)	0.36205 (15)	0.0355 (3)	0.0325 (7)	
H15	1.1545	0.3863	0.0949	0.039*	
C16	1.10554 (18)	0.29106 (15)	0.0505 (3)	0.0334 (8)	
H16	1.1333	0.2670	0.1203	0.040*	
C17	1.04975 (18)	0.50907 (14)	−0.1690 (3)	0.0321 (7)	
H17A	0.9892	0.5107	−0.1485	0.039*	
H17B	1.0557	0.4869	−0.2558	0.039*	
C18	1.08534 (18)	0.58205 (14)	−0.1706 (3)	0.0326 (7)	
H18A	1.0880	0.6005	−0.0802	0.039*	
H18B	1.0472	0.6127	−0.2227	0.039*	
C19	1.17292 (18)	0.58474 (14)	−0.2277 (3)	0.0339 (8)	
H19A	1.2107	0.5542	−0.1746	0.041*	
H19B	1.1700	0.5650	−0.3172	0.041*	
C20	1.21164 (18)	0.65730 (15)	−0.2341 (3)	0.0360 (8)	
H20A	1.2239	0.6742	−0.1442	0.043*	
H20B	1.1706	0.6900	−0.2753	0.043*	
C21	1.29262 (19)	0.65811 (16)	−0.3114 (3)	0.0384 (8)	
H21A	1.3319	0.6231	−0.2729	0.046*	
H21B	1.2792	0.6432	−0.4021	0.046*	
C22	1.3369 (2)	0.72806 (17)	−0.3155 (4)	0.0528 (10)	
H22A	1.3526	0.7424	−0.2253	0.063*	
H22B	1.2975	0.7637	−0.3517	0.063*	
C23	1.4159 (2)	0.7267 (2)	−0.3977 (4)	0.0689 (12)	
H23A	1.4527	0.6883	−0.3680	0.103*	
H23B	1.4458	0.7714	−0.3883	0.103*	
H23C	1.3999	0.7194	−0.4897	0.103*	
C24	0.73300 (17)	0.04826 (14)	0.2166 (3)	0.0266 (7)	
C25	0.65543 (18)	0.06211 (16)	0.1569 (3)	0.0376 (8)	
H25	0.6505	0.0617	0.0643	0.045*	
C26	0.58512 (19)	0.07654 (17)	0.2299 (3)	0.0417 (8)	
H26	0.5328	0.0862	0.1871	0.050*	
C27	0.59084 (18)	0.07690 (15)	0.3649 (3)	0.0321 (8)	
C28	0.66699 (18)	0.06206 (16)	0.4264 (3)	0.0380 (8)	
H28	0.6715	0.0615	0.5191	0.046*	
C29	0.73707 (19)	0.04797 (16)	0.3511 (3)	0.0363 (8)	
H29	0.7893	0.0378	0.3940	0.044*	
C30	0.5209 (2)	0.08959 (17)	0.5680 (3)	0.0431 (9)	
H30A	0.5677	0.1190	0.6024	0.052*	
H30B	0.5308	0.0407	0.5971	0.052*	
C31	0.4376 (2)	0.11602 (17)	0.6195 (3)	0.0469 (9)	
H31A	0.4344	0.1043	0.7134	0.056*	
H31B	0.3910	0.0914	0.5731	0.056*	
C32	0.42590 (19)	0.19494 (16)	0.6025 (3)	0.0435 (9)	
H32A	0.4785	0.2189	0.6308	0.052*	
H32B	0.4164	0.2052	0.5086	0.052*	
C33	0.35324 (19)	0.22487 (16)	0.6787 (3)	0.0430 (9)	
H33A	0.3588	0.2096	0.7709	0.052*	
H33B	0.2997	0.2057	0.6425	0.052*	
C34	0.3499 (2)	0.30462 (17)	0.6739 (4)	0.0500 (9)	
H34A	0.4052	0.3234	0.7034	0.060*	
H34B	0.3404	0.3195	0.5821	0.060*	
C35	0.2822 (2)	0.33668 (17)	0.7564 (4)	0.0568 (11)	
H35A	0.2896	0.3201	0.8476	0.068*	
H35B	0.2264	0.3206	0.7237	0.068*	
C36	0.2846 (3)	0.41618 (19)	0.7547 (5)	0.0884 (15)	
H36A	0.3389	0.4324	0.7906	0.133*	
H36B	0.2390	0.4346	0.8077	0.133*	
H36C	0.2775	0.4329	0.6646	0.133*	

### Atomic displacement parameters (Å^2^)

**Table:**

	*U*^11^	*U*^22^	*U*^33^	*U*^12^	*U*^13^	*U*^23^
Ni1	0.0215 (3)	0.0189 (3)	0.0279 (3)	−0.0005 (2)	0.0057 (2)	0.0001 (3)
N1	0.0224 (13)	0.0219 (13)	0.0262 (14)	0.0005 (11)	0.0043 (10)	−0.0013 (11)
N2	0.0220 (13)	0.0215 (13)	0.0269 (14)	0.0000 (10)	0.0044 (11)	−0.0015 (11)
O1	0.0413 (13)	0.0198 (11)	0.0359 (13)	−0.0051 (9)	0.0003 (10)	0.0042 (9)
O2	0.0328 (12)	0.0430 (14)	0.0473 (15)	0.0041 (10)	0.0168 (11)	−0.0031 (11)
C1	0.0256 (16)	0.0231 (16)	0.0238 (17)	0.0005 (13)	0.0022 (13)	0.0022 (13)
C2	0.0277 (16)	0.0217 (16)	0.0237 (17)	0.0032 (13)	0.0014 (13)	0.0000 (13)
C3	0.0311 (17)	0.0199 (16)	0.0336 (18)	0.0022 (13)	0.0032 (14)	−0.0009 (13)
C4	0.0271 (17)	0.0261 (17)	0.038 (2)	0.0063 (14)	0.0090 (15)	−0.0013 (14)
C5	0.0225 (16)	0.0208 (16)	0.0302 (17)	0.0005 (13)	0.0040 (13)	−0.0022 (13)
C6	0.0218 (16)	0.0264 (17)	0.0295 (18)	−0.0008 (13)	0.0045 (13)	−0.0022 (14)
C7	0.0230 (16)	0.0245 (17)	0.0330 (18)	−0.0021 (13)	0.0067 (14)	0.0008 (13)
C8	0.0240 (16)	0.0315 (18)	0.043 (2)	−0.0019 (14)	0.0118 (15)	0.0010 (15)
C9	0.0307 (18)	0.0250 (17)	0.042 (2)	−0.0039 (14)	0.0105 (15)	0.0005 (15)
C10	0.0264 (16)	0.0224 (16)	0.0243 (16)	−0.0012 (13)	0.0040 (13)	−0.0002 (13)
C11	0.0245 (15)	0.0206 (15)	0.0308 (17)	−0.0011 (13)	0.0060 (13)	−0.0015 (13)
C12	0.0396 (19)	0.0265 (17)	0.0330 (18)	−0.0060 (14)	0.0016 (15)	−0.0026 (14)
C13	0.0415 (19)	0.0260 (17)	0.0321 (18)	−0.0024 (15)	−0.0036 (15)	0.0037 (14)
C14	0.0297 (17)	0.0214 (15)	0.0318 (18)	−0.0023 (13)	0.0058 (14)	0.0015 (14)
C15	0.0371 (18)	0.0258 (17)	0.0343 (19)	−0.0076 (14)	−0.0072 (15)	−0.0027 (14)
C16	0.0359 (18)	0.0281 (17)	0.0361 (19)	−0.0002 (14)	−0.0031 (15)	0.0068 (15)
C17	0.0352 (17)	0.0246 (17)	0.0366 (18)	0.0001 (14)	0.0020 (14)	0.0064 (15)
C18	0.0372 (18)	0.0242 (17)	0.0367 (19)	0.0024 (14)	0.0072 (15)	0.0033 (14)
C19	0.0361 (18)	0.0227 (17)	0.043 (2)	−0.0001 (14)	0.0035 (15)	0.0013 (15)
C20	0.0364 (19)	0.0295 (17)	0.042 (2)	−0.0007 (15)	0.0066 (16)	0.0028 (15)
C21	0.041 (2)	0.0336 (19)	0.041 (2)	−0.0012 (16)	0.0039 (16)	0.0004 (16)
C22	0.053 (2)	0.037 (2)	0.070 (3)	−0.0073 (18)	0.025 (2)	0.0005 (19)
C23	0.064 (3)	0.063 (3)	0.081 (3)	−0.017 (2)	0.034 (2)	0.001 (2)
C24	0.0243 (16)	0.0194 (15)	0.0365 (19)	0.0005 (12)	0.0073 (14)	−0.0004 (13)
C25	0.0309 (18)	0.045 (2)	0.038 (2)	0.0039 (16)	0.0093 (15)	0.0022 (16)
C26	0.0257 (18)	0.051 (2)	0.049 (2)	0.0065 (15)	0.0046 (16)	0.0006 (18)
C27	0.0255 (17)	0.0257 (17)	0.046 (2)	0.0032 (13)	0.0144 (15)	−0.0016 (15)
C28	0.0343 (19)	0.046 (2)	0.034 (2)	0.0044 (16)	0.0071 (16)	−0.0030 (16)
C29	0.0246 (17)	0.043 (2)	0.041 (2)	0.0055 (15)	0.0044 (15)	−0.0006 (16)
C30	0.045 (2)	0.037 (2)	0.049 (2)	0.0030 (16)	0.0177 (18)	−0.0038 (17)
C31	0.043 (2)	0.041 (2)	0.058 (2)	0.0002 (17)	0.0284 (18)	−0.0068 (18)
C32	0.038 (2)	0.037 (2)	0.056 (2)	−0.0019 (16)	0.0214 (17)	−0.0056 (17)
C33	0.0360 (19)	0.0348 (19)	0.059 (2)	−0.0013 (16)	0.0176 (17)	−0.0069 (17)
C34	0.046 (2)	0.038 (2)	0.066 (3)	0.0018 (17)	0.0165 (19)	−0.0047 (19)
C35	0.045 (2)	0.040 (2)	0.086 (3)	0.0044 (17)	0.015 (2)	−0.013 (2)
C36	0.091 (3)	0.043 (3)	0.132 (4)	0.012 (2)	0.034 (3)	−0.014 (3)

### Geometric parameters (Å, °)

**Table:**

Ni1—N1	1.946 (2)	C19—H19A	0.9900
Ni1—N1^i^	1.946 (2)	C19—H19B	0.9900
Ni1—N2^i^	1.951 (2)	C20—C21	1.525 (4)
Ni1—N2	1.951 (2)	C20—H20A	0.9900
N1—C2	1.387 (3)	C20—H20B	0.9900
N1—C5	1.391 (3)	C21—C22	1.508 (4)
N2—C10	1.391 (3)	C21—H21A	0.9900
N2—C7	1.392 (3)	C21—H21B	0.9900
O1—C14	1.371 (3)	C22—C23	1.526 (4)
O1—C17	1.438 (3)	C22—H22A	0.9900
O2—C27	1.376 (3)	C22—H22B	0.9900
O2—C30	1.430 (4)	C23—H23A	0.9800
C1—C10^i^	1.378 (4)	C23—H23B	0.9800
C1—C2	1.388 (4)	C23—H23C	0.9800
C1—C11	1.503 (4)	C24—C29	1.378 (4)
C2—C3	1.428 (4)	C24—C25	1.389 (4)
C3—C4	1.337 (4)	C25—C26	1.384 (4)
C3—H3	0.9500	C25—H25	0.9500
C4—C5	1.428 (4)	C26—C27	1.383 (4)
C4—H4	0.9500	C26—H26	0.9500
C5—C6	1.380 (4)	C27—C28	1.381 (4)
C6—C7	1.382 (4)	C28—C29	1.394 (4)
C6—C24	1.502 (4)	C28—H28	0.9500
C7—C8	1.422 (4)	C29—H29	0.9500
C8—C9	1.343 (4)	C30—C31	1.520 (4)
C8—H8	0.9500	C30—H30A	0.9900
C9—C10	1.437 (4)	C30—H30B	0.9900
C9—H9	0.9500	C31—C32	1.525 (4)
C10—C1^i^	1.378 (4)	C31—H31A	0.9900
C11—C12	1.378 (4)	C31—H31B	0.9900
C11—C16	1.387 (4)	C32—C33	1.518 (4)
C12—C13	1.388 (4)	C32—H32A	0.9900
C12—H12	0.9500	C32—H32B	0.9900
C13—C14	1.380 (4)	C33—C34	1.522 (4)
C13—H13	0.9500	C33—H33A	0.9900
C14—C15	1.387 (4)	C33—H33B	0.9900
C15—C16	1.377 (4)	C34—C35	1.510 (4)
C15—H15	0.9500	C34—H34A	0.9900
C16—H16	0.9500	C34—H34B	0.9900
C17—C18	1.502 (4)	C35—C36	1.516 (5)
C17—H17A	0.9900	C35—H35A	0.9900
C17—H17B	0.9900	C35—H35B	0.9900
C18—C19	1.521 (4)	C36—H36A	0.9800
C18—H18A	0.9900	C36—H36B	0.9800
C18—H18B	0.9900	C36—H36C	0.9800
C19—C20	1.516 (4)		
			
N1—Ni1—N1^i^	179.996 (1)	C21—C20—H20A	109.1
N1—Ni1—N2^i^	89.88 (9)	C19—C20—H20B	109.1
N1^i^—Ni1—N2^i^	90.13 (9)	C21—C20—H20B	109.1
N1—Ni1—N2	90.13 (9)	H20A—C20—H20B	107.9
N1^i^—Ni1—N2	89.87 (9)	C22—C21—C20	115.0 (3)
N2^i^—Ni1—N2	180.0	C22—C21—H21A	108.5
C2—N1—C5	103.8 (2)	C20—C21—H21A	108.5
C2—N1—Ni1	128.28 (19)	C22—C21—H21B	108.5
C5—N1—Ni1	127.87 (18)	C20—C21—H21B	108.5
C10—N2—C7	104.0 (2)	H21A—C21—H21B	107.5
C10—N2—Ni1	128.07 (18)	C21—C22—C23	112.9 (3)
C7—N2—Ni1	127.92 (18)	C21—C22—H22A	109.0
C14—O1—C17	117.2 (2)	C23—C22—H22A	109.0
C27—O2—C30	116.9 (2)	C21—C22—H22B	109.0
C10^i^—C1—C2	122.2 (2)	C23—C22—H22B	109.0
C10^i^—C1—C11	119.2 (3)	H22A—C22—H22B	107.8
C2—C1—C11	118.6 (2)	C22—C23—H23A	109.5
C1—C2—N1	125.6 (2)	C22—C23—H23B	109.5
C1—C2—C3	123.3 (3)	H23A—C23—H23B	109.5
N1—C2—C3	110.9 (2)	C22—C23—H23C	109.5
C4—C3—C2	107.2 (3)	H23A—C23—H23C	109.5
C4—C3—H3	126.4	H23B—C23—H23C	109.5
C2—C3—H3	126.4	C29—C24—C25	117.6 (3)
C3—C4—C5	107.4 (3)	C29—C24—C6	119.8 (3)
C3—C4—H4	126.3	C25—C24—C6	122.6 (3)
C5—C4—H4	126.3	C24—C25—C26	121.2 (3)
C6—C5—N1	125.8 (2)	C24—C25—H25	119.4
C6—C5—C4	123.5 (3)	C26—C25—H25	119.4
N1—C5—C4	110.6 (2)	C27—C26—C25	120.2 (3)
C5—C6—C7	122.5 (3)	C27—C26—H26	119.9
C5—C6—C24	118.7 (3)	C25—C26—H26	119.9
C7—C6—C24	118.7 (3)	O2—C27—C28	124.6 (3)
C6—C7—N2	125.5 (3)	O2—C27—C26	115.9 (3)
C6—C7—C8	123.7 (3)	C28—C27—C26	119.5 (3)
N2—C7—C8	110.8 (2)	C27—C28—C29	119.3 (3)
C9—C8—C7	107.6 (3)	C27—C28—H28	120.4
C9—C8—H8	126.2	C29—C28—H28	120.4
C7—C8—H8	126.2	C24—C29—C28	122.1 (3)
C8—C9—C10	106.9 (3)	C24—C29—H29	119.0
C8—C9—H9	126.5	C28—C29—H29	119.0
C10—C9—H9	126.5	O2—C30—C31	108.7 (3)
C1^i^—C10—N2	125.8 (3)	O2—C30—H30A	109.9
C1^i^—C10—C9	123.6 (3)	C31—C30—H30A	109.9
N2—C10—C9	110.6 (2)	O2—C30—H30B	109.9
C12—C11—C16	117.3 (3)	C31—C30—H30B	109.9
C12—C11—C1	119.9 (3)	H30A—C30—H30B	108.3
C16—C11—C1	122.7 (3)	C30—C31—C32	113.1 (3)
C11—C12—C13	122.5 (3)	C30—C31—H31A	109.0
C11—C12—H12	118.8	C32—C31—H31A	109.0
C13—C12—H12	118.8	C30—C31—H31B	109.0
C14—C13—C12	119.3 (3)	C32—C31—H31B	109.0
C14—C13—H13	120.4	H31A—C31—H31B	107.8
C12—C13—H13	120.4	C33—C32—C31	113.9 (3)
O1—C14—C13	124.9 (3)	C33—C32—H32A	108.8
O1—C14—C15	116.1 (3)	C31—C32—H32A	108.8
C13—C14—C15	119.1 (3)	C33—C32—H32B	108.8
C16—C15—C14	120.7 (3)	C31—C32—H32B	108.8
C16—C15—H15	119.6	H32A—C32—H32B	107.7
C14—C15—H15	119.6	C32—C33—C34	112.7 (3)
C15—C16—C11	121.1 (3)	C32—C33—H33A	109.1
C15—C16—H16	119.4	C34—C33—H33A	109.1
C11—C16—H16	119.4	C32—C33—H33B	109.1
O1—C17—C18	108.4 (2)	C34—C33—H33B	109.1
O1—C17—H17A	110.0	H33A—C33—H33B	107.8
C18—C17—H17A	110.0	C35—C34—C33	114.3 (3)
O1—C17—H17B	110.0	C35—C34—H34A	108.7
C18—C17—H17B	110.0	C33—C34—H34A	108.7
H17A—C17—H17B	108.4	C35—C34—H34B	108.7
C17—C18—C19	112.5 (2)	C33—C34—H34B	108.7
C17—C18—H18A	109.1	H34A—C34—H34B	107.6
C19—C18—H18A	109.1	C34—C35—C36	112.3 (3)
C17—C18—H18B	109.1	C34—C35—H35A	109.1
C19—C18—H18B	109.1	C36—C35—H35A	109.1
H18A—C18—H18B	107.8	C34—C35—H35B	109.1
C20—C19—C18	115.0 (2)	C36—C35—H35B	109.1
C20—C19—H19A	108.5	H35A—C35—H35B	107.9
C18—C19—H19A	108.5	C35—C36—H36A	109.5
C20—C19—H19B	108.5	C35—C36—H36B	109.5
C18—C19—H19B	108.5	H36A—C36—H36B	109.5
H19A—C19—H19B	107.5	C35—C36—H36C	109.5
C19—C20—C21	112.3 (2)	H36A—C36—H36C	109.5
C19—C20—H20A	109.1	H36B—C36—H36C	109.5

Symmetry codes: (i) −*x*+2, −*y*, −*z*.
